# Bcl-2 and *β*_1_-integrin predict survival in a tissue microarray of small cell lung cancer

**DOI:** 10.1038/sj.bjc.6605950

**Published:** 2010-11-09

**Authors:** M H Lawson, N M Cummings, D M Rassl, S L Vowler, M Wickens, W J Howat, J D Brenton, G Murphy, R C Rintoul

**Affiliations:** 1 Cancer Research UK Cambridge Research Institute, Li Ka Shing Centre, Robinson Way, Cambridge, CB2 0RE, UK; 2 Department of Thoracic Oncology, Papworth Hospital NHS Foundation Trust, Papworth Everard, Cambridgeshire, CB23 3RE, UK; 3 Department of Oncology, University of Cambridge, Cambridgeshire, UK

**Keywords:** small cell lung cancer, tissue microarray, Bcl-2, *β*_1_-integrin

## Abstract

**Introduction:**

Survival in small cell lung cancer (SCLC) is limited by the development of chemoresistance. Factors associated with chemoresistance *in vitro* have been difficult to validate *in vivo*. Both Bcl-2 and *β*_1_-integrin have been identified as *in vitro* chemoresistance factors in SCLC but their importance in patients remains uncertain. Tissue microarrays (TMAs) are useful to validate biomarkers but no large TMA exists for SCLC. We designed an SCLC TMA to study potential biomarkers of prognosis and then used it to clarify the role of both Bcl-2 and *β*_1_-integrin in SCLC.

**Methods:**

A TMA was constructed consisting of 184 cases of SCLC and stained for expression of Bcl-2 and *β*_1_-integrin. The slides were scored and the role of the proteins in survival was determined using Cox regression analysis. A meta-analysis of the role of Bcl-2 expression in SCLC prognosis was performed based on published results.

**Results:**

Both proteins were expressed at high levels in the SCLC cases. For Bcl-2 (*n*=140), the hazard ratio for death if the staining was weak in intensity was 0.55 (0.33–0.94, *P*=0.03) and for *β*_1_-integrin (*n*=151) was 0.60 (0.39–0.92, *P*=0.02). The meta-analysis showed an overall hazard ratio for low expression of Bcl-2 of 0.91(0.74–1.09).

**Conclusions:**

Both Bcl-2 and *β*_1_-integrin are independent prognostic factors in SCLC in this cohort although further validation is required to confirm their importance. A TMA of SCLC cases is feasible but challenging and an important tool for biomarker validation.

Despite initial good response rates to chemotherapy (Amarasena *et al*, 2008), the overall 5-year survival rate for small cell lung cancer (SCLC) is only 5% (Hann and Rudin, 2007). This paradoxically poor survival rate is due to the fact that patients almost invariably relapse with disease that is resistant to chemotherapy; relapse rates of up to 95% have been reported (Huisman *et al*, 1999). Treatment refractory disease is the major factor that limits improvement in survival rates for this type of lung cancer. As very few patients with SCLC have surgical resection, tumour samples available for research are usually restricted to small biopsy specimens. Consequently, much of the study of SCLC biology has been accomplished using a limited repertoire of cell lines generated from tumours, frequently derived after initial treatment (Carney *et al*, 1985). High expression of the cell adhesion protein *β*_1_-integrin and the anti-apoptotic protein Bcl-2 have both been associated with increased cell survival in *in vitro* models of SCLC (Sethi *et al*, 1999; Pardo *et al*, 2002; Sartorius and Krammer, 2002) but *in vivo* confirmation of an important role in patients with SCLC is sparse. For *β*_1_-integrin, there is evidence from a small Japanese study that low expression is associated with a better outcome (Oshita *et al*, 2002). For Bcl-2, no relationship between expression levels and survival has been confirmed in SCLC (Martin *et al*, 2003; Ilievska Poposka *et al*, 2008). Despite this, trials of treatment targeted at Bcl-2 in SCLC are in progress (Hann *et al*, 2008; Rudin *et al*, 2008; Shoemaker *et al*, 2008).

Tissue microarrays (TMAs) were developed to allow high-throughput analysis of protein expression in tumour tissues (Kononen *et al*, 1998). The use of TMAs to validate biomarkers of outcome in tumour tissue is well established (Hassan *et al*, 2008). Here, we report the first SCLC TMA for biomarker validation, which we have used to investigate a role for both *β*_1_-integrin and Bcl-2 expression in the survival of patients with SCLC.

## Materials and methods

Ethical approval for the use of samples and data collection was granted by the local Research Ethics Committee. All cases of SCLC diagnosed at Papworth Hospital (Cambridge, UK) between 1998 and 2005 were identified from hospital records and the formalin fixed, paraffin-embedded biopsy tissue samples were retrieved from the pathology department store along with their associated histology slides. Clinical data relating to the cases was compiled from a database held at Papworth Hospital. Survival data were confirmed from records of deaths or via the family doctor of the patient concerned.

Histology slides were examined, and areas of tumour tissue were marked. Whenever possible, at least two areas of tumour tissue were marked on each slide. In some cases, multiple blocks taken from the same tumour were available, and a slide from each block was included. The marked slides were scanned onto the Ariol SL50 system (Genetix, Gateshead, UK) for ease of comparison with the cut surfaces of the paraffin blocks and 0.6 mm cores of the marked areas were taken using a Beecher Manual Tissue Arrayer 1 (Beecher Instruments, Sun Prairie, WI, USA). The cores (including marker cores for orientation and cores of control material from patients without cancer and from areas of normal appearing tissue from the SCLC tissue blocks) were assembled into a TMA and cut across four slides to enable full representation.

Slides were stained on a BondMax Autostainer (Leica, Milton Keynes, UK). Antigen retrieval was performed at 100°C in Bond ER2 diluent, followed by a 15-min incubation with primary antibody at room temperature, 8-min incubation using a polymer secondary system (Leica) followed by developing with Diaminobenzidine using copper enhancement. Haematoxylin counterstaining was performed automatically on the Bond system, and finally, the slides were dehydrated, cleared and mounted using a Leica ST5020 attached coverslipper CV5030 (Leica). *β*_1_-Integrin was detected using CD29 antibody diluted 1 : 100 (Novocastra, Newcastle, UK), and Bcl-2 was detected using Clone 124 antibody diluted 1 : 200 (Dako, Glostrup, Denmark). The slides were scanned onto the Ariol system for analysis.

**Figure 1:**
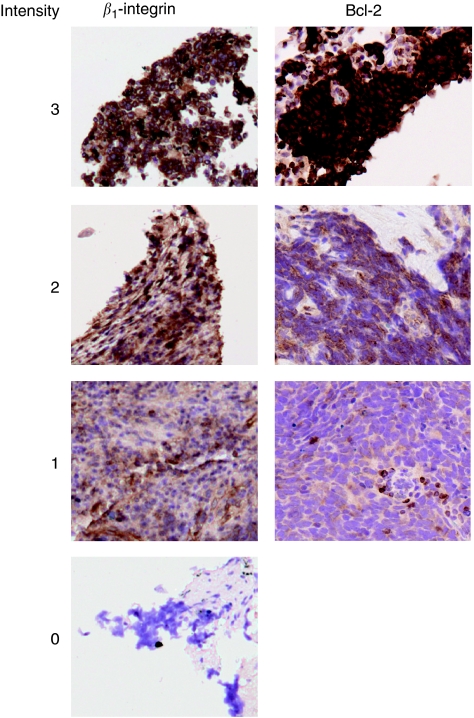
Staining pattern of SCLC cores for *β*_1_-integrin and Bcl-2. The TMAs were stained and scanned onto the Ariol system. Consensus scoring was performed by two blinded observers. Scores were assigned based on staining intensity. The images shown are from the Ariol system and were cropped to size using Photoshop.

Scoring was performed simultaneously by two observers (one of whom was a senior pulmonary histopathologist) blinded to the clinical data, and a consensus score reached for each tissue core. For each core and for each antibody, both extent and intensity of tumour staining were evaluated on a scale from 0 to 3 after the method by Allred *et al* (1998). For *β*_1_-integrin, extent was scored with 0 if <10% of tumour cells positive, 1=10–25%, 2=25–50% and 3 if >50% (Oshita *et al*, 2002; Yao *et al*, 2007). For Bcl-2, extent was scored with 0 if <10% of tumour cells positive, 1=10–50%, 2=50–75% and 3 if >75%. For both proteins, intensity was scored as 0=no staining, 1=low-intensity staining, 2=medium-intensity staining and 3=high-intensity staining (Figure 1). Data analysis was performed with SPSS v17.0 (SPSS Inc., Woking, UK). Univariable survival analysis used the log rank test (LR), and multivariable analysis was performed by Cox regression using a forward stepwise model based on likelihood ratios. Results were displayed as Kaplan–Meier plots. Categorical outcomes were analysed using the *χ*^2^-test.

Initial analysis used previously published criteria to define cases as having positive or negative expression of each protein, with a cutoff of 10% of cells with staining for Bcl-2 (Martin *et al*, 2003) and 25% for *β*_1_-integrin (Oshita *et al*, 2002). Further analysis was based on plotting survival for each extent or intensity score and examining the survival curves to see if the cases could reasonably be dichotomised into good- and poor-prognosis groups.

A meta-analysis was performed of studies, which compared Bcl-2 staining by immunohistochemistry with survival for patients with SCLC. This was based on eight published studies along with the results from the current study (Kaiser *et al*, 1996; Takayama *et al*, 1996; Dingemans *et al*, 1999; Maitra *et al*, 1999; Zereu *et al*, 2003; Paik *et al*, 2006; Ilievska Poposka *et al*, 2008; Lee *et al*, 2008). Hazard ratios for death if Bcl-2 negative were taken from multivariable analysis in the original paper. If the original publication did not contain these data, it was calculated from the data presented by Martin *et al* (2003) or calculated using the indirect method by Parmar *et al* (1998). A summary hazard ratio was calculated, and a forest plot was created using the R package ‘rmeta’ (Lumley, 2009).

## Results

In all, 203 cases of SCLC diagnosed at Papworth Hospital between 1998 and 2005 were identified from the Pathology department archive. Of these 203 cases, 184 had tissue on at least one level of the TMA. Overall survival data were complete to the end of 2008. Characteristics of the cohort are shown in Table 1. Baseline demographic characteristics that were found to be significant prognostic factors in univariable analysis were stage, age, performance score and first-line treatment. Cox regression analysis was performed to identify independent prognostic variables. For each variable, a log minus log plot was created and confirmed proportionality of hazards. The Cox regression model had 153 patient events in 166 patients with a complete data set and included stage and first-line treatment as independent variables (*P*<0.0005). Median survival was 13.5 months (9.8, 17.2 months) for patients with limited stage disease and 5.5 months (3.9, 7.2 months) for extensive stage.

For Bcl-2, 140 cases, of the possible 184 cases present on the whole TMA, had sufficient stained tumour tissue to evaluate staining at the level of the TMA used. There was no evidence of a difference in baseline characteristics or in survival between cases whose staining could be assessed and the remaining cases. There were 58 cases with more than one core of tumour on the TMA; 49 cases had 2 cores. Where more than one core was present and the scores were not identical, the modal score was taken for each tumour; this happened in seven cases. Where this was not possible, the highest score was taken and this occurred in 27 cases. In the remaining 24 cases, all the cores were scored identically.

**Figure 2:**
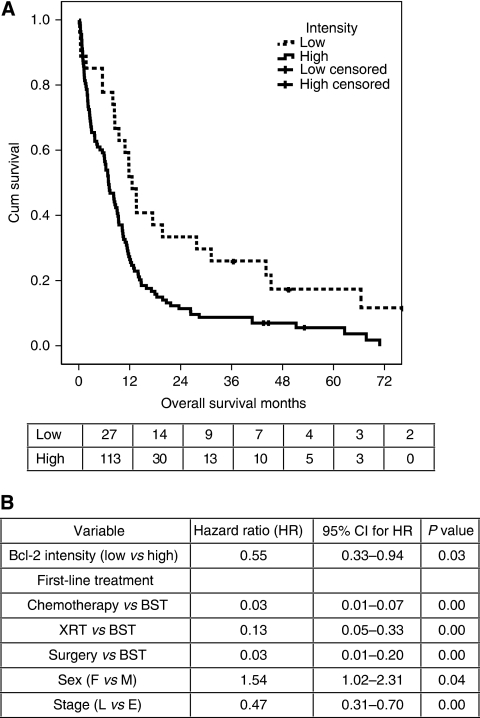
Analysis of the role of Bcl-2 staining in survival in SCLC. (**A**) Overall survival analysis was performed based on the patients who had Bcl-2 staining scored on the TMA (*n*=140). Variables were assessed by Kaplan–Meier analysis and survival curves were plotted. The variables that were significant in the whole patient cohort remained significant in this cohort. The Kaplan–Meier plot for Bcl-2 intensity (*n*=140) is shown, *P*=0.004 by log rank test. The numbers of patients at risk in each group at each time point are shown below the plot. (**B**) Variables that were positive in univariable analysis were used for Cox regression analysis. A multivariable model was constructed using a forward stepwise method based on likelihood ratios. Four variables were independent predictors of outcome; stage, sex, first-line treatment and intensity of staining for Bcl-2 (*P*=0.03, *n*=117). Age at diagnosis and PS were rejected. XRT=radiotherapy; BST=best supportive care; L=limited; E=extensive; HR=hazard ratio; CI=confidence interval.

The majority of cases had extensive staining for Bcl-2. There was only one case where <10% of cells were stained and 82% (115 out of 140) of evaluated cases had >50% cells positive. No differences in survival were detectable based on differences in staining extent. Survival analysis revealed that the most informative categorisation by intensity was with a cutoff between low (intensity score 1) and medium intensity (score 2). Therefore, cases were reclassified as either high-intensity staining (score 2 or 3) or low-intensity staining. Analysis of survival showed that patients with low-intensity staining survived for longer (LR=8.09, *P*=0.004, Figure 2A), and this relationship was maintained in the sub-group treated with chemotherapy (*n*=95, LR=5.22, *P*=0.022). Cox regression analysis with age (as a continuous variable), sex, stage, performance score, first-line treatment and staining intensity as the input variables confirmed a significant role for Bcl-2 as an independent predictor of survival in this cohort (Figure 2B). The hazard ratio for death in patients with low-intensity staining was 0.55 (95% CI=0.33, 0.94, *P*=0.03, *n*=117). There was no evidence of an effect of Bcl-2 staining on progression-free survival, and Bcl-2 status was not predictive of response to chemotherapy.

**Figure 3:**
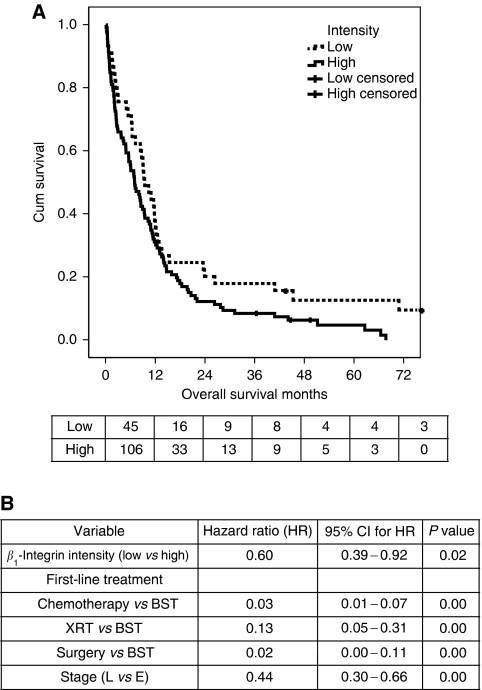
Analysis of the role of *β*_1_-integrin staining in survival in SCLC. (**A**) Overall survival analysis was performed based on the patients who had CD29 (*β*_1_-integrin) staining scored on the TMA (*n*=151). Variables were assessed by Kaplan–Meier analysis and survival curves were plotted. The variables that were significant in the whole patient cohort remained significant in this cohort. The Kaplan–Meier plot for *β*_1_-integrin intensity (*n*=151) is shown, *P*=0.05 by log rank test. The numbers of patients at risk in each group at each time point are shown below the plot. (**B**) Variables that were positive in univariable analysis were used for Cox regression analysis. A multivariable model was constructed using a forward stepwise method based on likelihood ratios. Three variables were independent predictors of outcome; stage, first-line treatment and intensity of staining for *β*_1_-integrin (*P*=0.02, *n*=121). Age at diagnosis, sex and PS were rejected. XRT=radiotherapy; BST=best supportive care; L=limited; E=extensive; HR=hazard ratio; CI=confidence interval.

Only 151 cases had tumour tissue available to analyse for *β*_1_-integrin staining at the TMA level used. Baseline characteristics and survival were not different for those patients from the whole cohort who had cores stained for *β*_1_-integrin in comparison to those who did not. For *β*_1_-integrin, differences in staining extent were not predictive of survival and only 8.6% (13 out of 151) of cases scored had <50% of cells stained. On the basis of the Kaplan–Meier plot of all categories of *β*_1_-integrin staining intensity, intensity was dichotomised into those with high-intensity staining (score of 3) and those with lower-intensity staining (score 0–2). Using this classification, high-intensity staining carried a worse prognosis (LR=3.85, *P*=0.05; Figure 3A). In Cox regression analysis, only stage, first-line treatment and staining intensity were retained in the model (*n*=121; Figure 3B). Patients with a low intensity of staining had a hazard ratio of death of 0.60 (0.39, 0.92) compared with patients with high intensity (*P*=0.019). However, *β*_1_-integrin staining showed no evidence of being predictive of response and had no impact on progression-free survival in those treated with chemotherapy.

**Figure 4:**
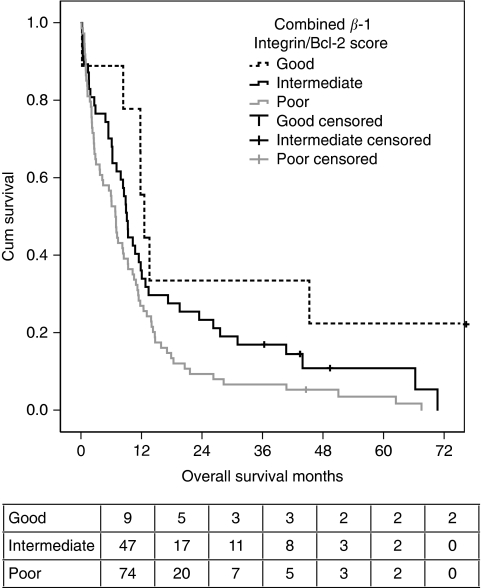
Combining results of *β*_1_-integrin staining and Bcl-2 staining identifies three groups with differing prognoses. Cases were grouped based on expression of *β*_1_-integrin and Bcl-2 into cases where (1) both proteins had high expression (*n*=74; predicted to have poor prognosis), (2) both had low expression (*n*=9; predicted to have good prognosis) and (3) those with discordant expression levels (*n*=47; predicted to have intermediate prognosis). Survival was plotted by the Kaplan–Meier method showing significant differences between the three groups (log rank=8.98; *P*=0.01). The numbers of patients at risk in each group at each time point are shown below the plot.

Data for survival based on intensity of *β*_1_-integrin staining and Bcl-2 staining were combined. Cases were categorised based on low or high intensity of staining for each antibody into three groups. The ‘good-prognosis’ group (*n*=9) had low-intensity staining for both antibodies, the ‘poor-prognosis’ group (*n*=74) had high-intensity staining for both antibodies and the ‘intermediate-prognosis’ group had discordant staining for the two antibodies with one low and one high intensity (*n*=47). Univariable survival analysis revealed that the good-prognosis group had a median survival of 12.5 months (10.29, 14.71) compared with 9.18 months (8.12, 10.24) for the intermediate group and 6.8 months (5.49, 8.06) in the poor-prognosis group (LR=8.98, *P*=0.011; Figure 4). In a Cox regression model based on the same variables as for the individual antibodies, the ‘good-prognosis’ group had a hazard ratio for death of 0.28 (0.11, 0.67), but the survival difference between the intermediate and poor groups was lost (*n*=108).

**Figure 5:**
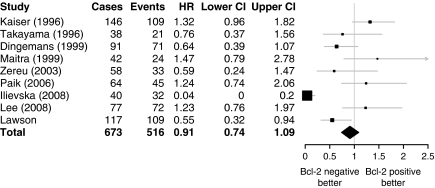
Forest plot of role of Bcl-2 in SCLC survival from the literature. Previous studies were combined in a meta-analysis with the results of the current study and a forest plot generated. The summary hazard ratio (HR) suggests better survival in Bcl-2-negative cases but does not reach significance. Box size is proportional to accuracy of estimate. CI=confidence interval.

The meta-analysis of the effect of Bcl-2 negativity on survival identified 516 deaths in a cohort of 673 cases, including the data presented in the current paper (Figure 5). The overall hazard ratio was 0.91, indicating better survival for patients negative for Bcl-2 staining but this was not statistically significant.

## Discussion

This paper describes the first dedicated SCLC TMA for biomarker discovery and validation to be reported. The cohort of patients studied is an unselected group of SCLC patients representative of the spectrum of disease seen and treated in the UK (El-Helw *et al*, 2008). Three groups have published data from a TMA with SCLC cases previously, but all used surgical samples of SCLC to construct the TMA, and this makes them unrepresentative of the clinical disease spectrum (Donati *et al*, 2004; Nitadori *et al*, 2006; Chiappori *et al*, 2010). The Japanese group included 14 surgically resected cases with 39 cases of large-cell neuroendocrine cancer on a TMA designed to compare the immunohistochemical staining patterns of the two diseases (Nitadori *et al*, 2006). An Italian group obtained tissue from 54 cases of SCLC treated by surgical resection over a 21-year period and looked at the accuracy of biomarker staining on the TMA in comparison to ‘whole sections’ of tissue (Donati *et al*, 2004). They found that in comparison to whole sections, the TMA had an accuracy of 87%, and the shortfall was attributed to small cores of tissue not representing the whole tumour well (Donati *et al*, 2004). They did not present data on the accuracy of the TMA compared with sections cut from biopsies, which like the TMA cores, may not represent the whole tumour accurately, and they did not look at the impact of staining in the TMA on clinical end points. Their conclusion was that a TMA could not replace examination of tissue sections in the diagnosis of SCLC. The most recent paper reported data from a commercially made TMA including 100 cases of SCLC (Chiappori *et al*, 2010) and described the pattern of specific marker expression using an automated analysis system. The origins of the tissue used for the TMA was unclear in terms of collection method, but they were probably surgical samples, and 23 of the 100 cases were mixed SCLC and other histologies (Chiappori *et al*, 2010; Shanghai Outdo Biotech Ltd.).

Traditionally, surgical specimens have provided the tissue cores for TMA construction, as they provide an easy to handle source of large quantities of tissue for most tumours. Small Cell Lung Cancer is a special case as resection is not part of the usual clinical management of patients. The biopsies used for diagnosis are small making them difficult to handle and frequently have <50% tumour in them (Coghlin *et al*, 2010). The reason analysis of prognostic factors in SCLC has not been studied using a TMA previously is almost certainly the technical difficulty of constructing a TMA from small biopsy samples. In the TMA constructed from surgical resections of SCLC, 13.4% of cores were lost from the donor block to the recipient TMA block (Donati *et al*, 2004). In the current TMA, 203 cases were identified, and 184 cases had tissue on the TMA at some level. The small biopsy samples are not perfectly aligned on the TMA and do not contain homogenous tumour samples. This means that at a given level of the TMA, tumour tissue may be present but then absent at deeper levels and present again at even deeper levels. This explains why the sample size was 151 cases for *β*_1_-integrin but only 140 for Bcl-2. However, there was no evidence of this dropout altering the characteristics of the study population in terms of demographics or survival. Dropout of cases must be tolerated as limiting analysis to surgically resected tumours makes the cohort unrepresentative of clinical disease. Dropout of cases is an important consideration when prospectively planning TMA construction to ensure an adequate sample size for the planned analyses. Practically, the small numbers of available cases in SCLC means that minimisation of dropout during construction of the TMA is paramount and requires a high degree of technical skill. Ideally, multiple cores of each tumour would be placed on the TMA to minimise misrepresentation, but this is not a realistic proposition in SCLC due to lack of sufficient tissue.

For both Bcl-2 and *β*_1_-integrin, there is good evidence that they can protect SCLC cells from chemotherapy-induced apoptosis *in vitro*. However, confirmation that this is important *in vivo* is lacking (Sethi *et al*, 1999; Sartorius and Krammer, 2002). Our primary data, which equals the largest previous study in terms of statistical power, found that low Bcl-2 expression was an independent predictor of improved prognosis in SCLC, which supports the hypothesis driving the study of the use of Bcl-2 antagonists in SCLC (Rudin *et al*, 2008; Shoemaker *et al*, 2008). The meta-analysis performed in this study, which builds on the work by Martin *et al* (2003), shows that no clear relationship between Bcl-2 expression and survival has yet been identified.

Low expression of *β*_1_-integrin was also shown to be an independent marker of better prognosis in the current study, confirming the work by Oshita *et al* (2002). This is consistent with the *in vitro* findings that extracellular matrix proteins can activate phosphatidylinositol-3-kinase/AKT dependent pro-survival pathways via binding to *β*_1_-integrin (Sethi *et al*, 1999). Phosphatidylinositol-3-kinase/AKT signalling is able to activate Bcl-2 and signalling via *β*_1_-integrin has been linked previously to the promotion of cell survival via Bcl-2 upregulation (Zhang *et al*, 1995; Vivanco and Sawyers, 2002). In the current study, combining the staining results for both proteins allowed the identification of a small subset of patients with a particularly good prognosis.

The definition of positive staining for each protein is of key importance in assessing the validity of the results obtained. Overall, protein expression in a tissue can be considered to have two aspects, extent of staining (the number of cells that exhibit staining) and intensity of staining (the amount of staining in positive cells). Either parameter, or both, or neither, may have prognostic relevance for a given protein in a specific situation. Extent is commonly used to define positive staining for the purposes of survival analysis as intensity of staining can be influenced by subtle differences in the staining process between different batches of sections processed. This problem does not occur when staining a TMA. For both Bcl-2 and *β*_1_-integrin, extent of staining is the criterion that has been used previously to define positive staining with a cutoff of 10% of tumour cells for Bcl-2 and 25% for *β*_1_-integrin. However, it is apparent from Figure 5 that the 10% cutoff, used in all the studies except those of Kaiser *et al* (1996) (50% cutoff) and Lee *et al* (2008) (both extent and intensity of staining), has not been validated, as it does not give consistent results and the 25% cutoff for *β*_1_-integrin has only been reported in a single cohort. In the current study, extent of staining was not found to be informative for survival but unbiased examination of survival by intensity of staining was. A plausible explanation for the lack of discrimination of staining extent compared with intensity in our study would be increased sensitivity of the staining methods for the detection of positive cells. Increased sensitivity may be due to better antigen retrieval, the use of automated staining or better signal amplification systems (such as the polymer secondary system we used). For Bcl-2, it is unlikely to be due to the primary antibody as the Clone 124 antibody is a standard, well validated, antibody that is used widely (Martin *et al*, 2003).

A survival advantage for patients with low levels of expression of both Bcl-2 and *β*_1_-integrin was demonstrated, but in both cases, the low expression group was relatively small. This leads to the possibility of the data being ‘over-fitted’ and the results an artefact. This and the caveats in this study relating to the *post hoc* definition of the groups with differing survival would both be addressed by replication in an independent cohort of samples. This is the first large SCLC TMA to be reported in the literature and was technically difficult to construct. Replication in an independent sample set is an important future challenge.

In summary, we have demonstrated that construction of a TMA for the validation of *in vitro* findings and biomarker studies is feasible in SCLC. We have used our TMA to confirm that both *β*_1_-integrin and Bcl-2 are independent markers of adverse prognosis in SCLC. For both biomarkers, we report the most statistically powerful analysis done in SCLC patients in a cohort treated in line with current best practice. The results highlight these two molecules as potentially useful targets for future work.

## Figures and Tables

**Table 1:** Characteristics of the patients in the whole cohort and those with cores stained for Bcl-2 and *β*_1_-integrin

	**TMA**	**Bcl-2**	***β*_1_-integrin**
Total cases	184	140	151
Sex (male %)	63	62	63
			
*Age (years)*			
Mean (s.d.)	67.17 (10.00)	67.27 (9.92)	67.34 (9.93)
			
*Stage* (%)			
Limited	44	44.5	45
Extensive	54	54.5	52
Unknown	2	1	3
			
*Performance score* (%)			
0, 1	53	57	53
⩾2	30	26.5	28.5
Unknown	17	16.5	18.5
			
*First-line treatment* (%)			
Chemotherapy	66	68	66
Radiotherapy	15	15	16
Surgery	2	2.5	3
Supportive care	12	11	10
Unknown	5	3.5	5

Abbreviations: TMA=tissue microarray; s.d.=standard deviation.
